# Osler-Weber-Rendu Disease Uncovered by Preeclampsia in a Case Report

**DOI:** 10.1155/2020/2746947

**Published:** 2020-03-03

**Authors:** Jamal Ouachaou, Hamza Mimouni, Mohammed Maarad, Yassine Mellagui, Asmae Oulad Amar, Houssam Bkiyar, Imane Kamaoui, Brahim Housni

**Affiliations:** ^1^Intensive Care Unit, Mohammed VI University Hospital Center, Faculty of Medicine and Pharmacy of Oujda, Mohammed I University, Oujda, Morocco; ^2^Radiology Department, Mohammed VI University Hospital Center, Faculty of Medicine and Pharmacy of Oujda, Mohammed I University, Oujda, Morocco

## Abstract

Osler-Weber-Rendu disease (OWRD), called hereditary hemorrhagic telangiectasia, is an uncommon genetic illness with the dominant autosomal transmission. It cannot be easily or quickly diagnosed because of both its infrequency and its various associated symptoms. As far as its symptoms are concerned, the patient experiences recurring epistaxis, mucocutaneous telangiectasia, and arteriovenous malformations that can lead to severe undesirable symptoms. In our case, we report a 32-year-old female that was diagnosed with postpartum preeclampsia and whose paraclinical examinations showed that she suffers from hereditary hemorrhagic telangiectasia disease. Management of OWRD includes systematic diagnosis of visceral arteriovenous malformations (AVMs) in regular intervals, measures to prevent complications, and symptomatic treatment.

## 1. Introduction

Hereditary Rendu-Osler disease (MRO) or hereditary hemorrhagic telangiectasia is a form of vascular dysplasia involving hemorrhagic symptoms, cutaneous mucosal telangiectasia, and visceral shunts with arteriovenous defects. It is pregnancy that contributes to the proliferation and growth of VAM. What causes this disease is the vessels' disorder (vasculogenesis), disruptive angiogenesis, and vessels' regulation (homeostasis). The basic lesion is the distension of the distal vessels (telangiectasia) which is marked by a hemorrhagic tendency when it is cutaneous or mucous. On the other hand, when located in an organ, this type of lesion is reflected in the installation of an arteriovenous shunt between the arterioles and the venules, which are dependent on the viscera where the lesion is installed [1].

This report describes the case of a 32-year-old woman who got diagnosed with postpartum preeclampsia and suffers from stem and sublingual telangiectasia. The investigations showed the presence of the liver's subcapsular hematoma and arteriovenous malformations in pulmonary and hepatic areas.

## 2. Observation

Our patient is a 32-year-old female from a low social class and married for 7 years. She has already lost her fetus after 34 AW 4 years ago, and she had already undergone miscarriage after 16 AW 2 years ago. One year after, she suffered from epistaxis two times, and she was put in medical reanimation after 15 days following her pregnancy. She gave birth to her baby boy vaginally. The progression of the disease was noticed two days after the installation of headache in helmets with epigastric pain in irradiating strips towards the right hypochondrium.

The physical examination revealed a high blood pressure that reaches TA = 150/80 mmHg in the right limb and 155/90 mmHg in the left one, FC = 90 bpm, labstix: 2 proteinuria crosses, and stable in respiration. Moreover, the patient has epigastric sensitivity and telangiectasia in both the trunk and the sublingual area.  Table 1 presents the main biological results;

In the aforementioned table, the postpartum preeclampsia' diagnosis was noted.

**Figure 1:**
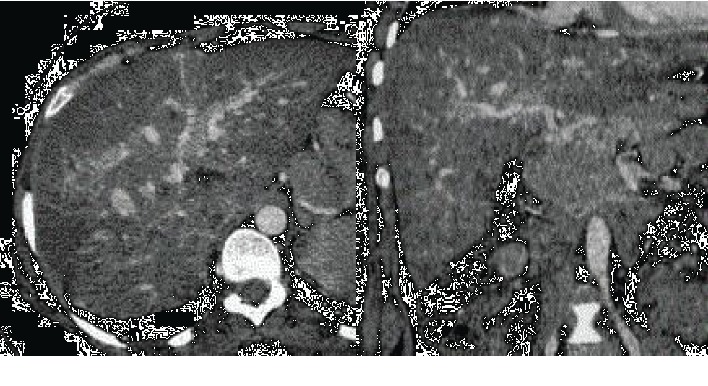
The transversal and sagittal section of the CT scan shows arteriovenous disorders in the liver.

**Figure 2:**
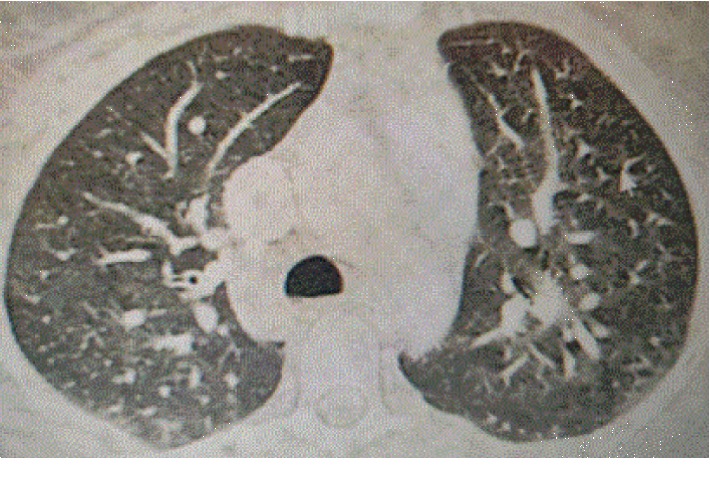
The transversal section of the CT scan shows arteriovenous pulmonary disorders.

An abdominal ultrasound scan was conducted to look for a subcapsular hematoma of the liver that returned in favour of a hypoechogenic lesion in segments VII and II of the liver and which was complemented by an angioscanner that showed vascular abnormalities in the lung and the liver (Figures 1 and 2). Encephalic MRI is positive.

Oesophagogastroduodenoscopy was conducted in favour of the following:

- erythematous and nodular antritis
- Antral millimetric telangiectasia lesion that bleeds
- Telangiectasic lesions scattered in both the bulb and the duodenum

Treatment is based on treating preeclampsia using oral antihypertensives (nicardipine 50 mg 2x/day) and proton pump inhibitors per bone 40 mg/day.

The patient's situation was improved after two days of treatment in that the pain disappeared.

## 3. Discussion

One of the first pioneers who had initiated the talk about this disease is the French dermatologist Henri Rendu in 1896. Following his discovery, in 1902, the Canadian doctor, William Osler, had elaborated on it more. Then, another English dermatologist called Frederick Parkes Weber provided enough information on it in 1907. In 1909, the American doctor named Hanes renamed this disease with HHT that stands for hereditary hemorrhagic telangiectasia.

Osler-Weber-Rendu disease is a multisystemic vascular dysplasia. The diagnosis is clinically based on the presence of spontaneous and recurrent epistaxis (occurring in the absence of blood coagulation disorders), multiple telangiectasias (lips, mouth, fingers, and nose), visceral sites, and prior presence in the family history that are relative to the first degree of the disease. According to the diagnostic criteria of the Curacao Consensus Conference (Table 2), the diagnosis of ORM is regarded as positive when at least three of the criteria are present [2]. The diagnosis of ORM is suspected when two criteria are observed, including the presence of pulmonary arteriovenous malformations.

Pulmonary arteriovenous abnormalities of ORM are more common among women (sex ratio 1.5 : 1). Since the predominance of the disease is age-related, their penetrance increases over the life cycle. There are about 10% of pulmonary arteriovenous defects that are identified during childhood or adolescence, and then, its rate increases starting from the second decade to approximately 30% [3].

About 70% of pulmonary AVMs are part of the ORM. They are more often multiple (35-65% of cases) [3–6] and bilateral [7]. Also, they would be more often related to neurological complications than isolated MAVPs that occur in the absence of an MRO.

### 3.1. Liver Arteriovenous Malformations

Liver disease is prevalent in 41-78% patients suffering from ORM, but the majorities show no symptoms [8, 9].

There are three shunt types that can occur together. For instance, there are shunts between the hepatic artery and the hepatic vein, the hepatic artery and the portal vein, and the portal vein and the hepatic vein.

These intrahepatic shunts are responsible for the development of cardiac dysfunction, portal hypertension, and biliary ischemia. The best test that can be conducted to screen hepatic AVMs is abdominal Doppler ultrasound or hepatic MRI [10].

### 3.2. Special Cases of Pregnant Women

To be pregnant while suffering from ORM is risky even if there are no pregnancy-related complications for most cases. For any patient desiring to become pregnant, she should receive multidisciplinary preconception guidance and screening tests especially if the patient is diagnosed with Osler rendering disease.

Different types of procedures can be offered to patients depending on the degree and frequency of epistaxis. For instance, FLOSEAL hemostatic foam, used in local application, has shown positive results and decreases the use of more invasive treatments [12]. Selective embolization, arterial ligation, endonasal coagulation of telangiectasias using Nd:YAG or KTP lasers, and bipolar electrical cauterization are sort of procedures that are often involved. In the case of severe epistaxis, performing skin transplantation (Saunders' operation) or closing the nasal cavities (Young's operation) can be recommended.

The treatment of hormones using estrogen-progestin or antiestrogen (tamoxifen) is proposed in some cases but remains controversial, especially in the case of men and postmenopausal women due to their undesirable effects [13].

Bevacizumab which is an anti-angiogenic treatment for some tumors, and tranexamic acid which is an antifibrinolytic, have shown some usefulness in reducing the frequency and severity of epistaxis in ORM, but are not yet used in clinical practices [14, 15].

In our case study, the patient exhibited neglected epistaxis and is presented in a postpartum preeclampsia table. The clinical investigation shows signs of telangiectasia preeclampsia. Other paraclinical examinations were in favour of arteriovenous liver and lung malformations. However, encephalic MRI has shown no abnormalities.

In our example, the patient is asymptomatic and the treatment was symptomatic.

After a six-month control of the patient's situation, it has been found that she has experienced two episodes of epistaxis treated symptomatically (tranexamic acid 500 mg 3x/day). The clinical examination found that there is the persistence of sublingual and trunk telangiectasia without extension.

## 4. Conclusion and “Take-Away”

MRO is marked by spontaneous epistaxis, mucocutaneous telangiectasia, and visceral arteriovenous defects (lung, liver, digestive tract, and brain).

The therapy includes a screening of visceral arteriovenous malformations and treatments to prevent their complications.

## 5. Patient's Perspective

The patient expressed her satisfaction with the treatment and was connected to the hepatic gastroenterology consultation for possible medical follow-up.

## Figures and Tables

**Table 1:** The main biological results.

Creatinine (*μ*mol/L)	30
Urea (mmol/L)	2
Proteinuria of 24 h g/L	232
ASAT	55
ALAT	45
Lipase	61
Hemoglobin (g/dL)	9.7
Platelet (G/L)	277
Prothrombin rate (%)	66
Fibrinogen (g/L)	3.8

**Table 2:** Curaçao Diagnostic Criteria, 1997 [11].

Epistaxis Spontaneous, repeated, and irregular nasal bleeding which can cause to chronic anemia
Telangiectasias Terminal dilations of blood vessels or lesions, cutaneous (located on the lips, fingers, face, hands, and feet) and mucous (inner surface of the lips, tongue, palate, nasal, and digestive mucosa)
Family affectedness The existence of at least one of the parents who suffer from the first degree of MRO. Due to the dominant autosomal aspect, this criterion is confirmed in more than 86% cases. Penetrance is almost complete at the age of 50. There are many variations in expressivity ranging from neonatal to asymptomatic adult forms
Arteriovenous abnormalities (AVM) The visceral disease can replace one of the three previous signs in the positive diagnosis. The location of the MAVs can be as follows: (i) Pulmonary, 30-50% of patients (ii) Hepatic, 30-80% of patients (iii) Neurological, 8-25% of patients (iv) In the digestive system
